# 

**Published:** 1895-06-08

**Authors:** 


**Notices of Births, Deaths, and Marriages** are inserted in this column at a charge of 2s. 6d. for an announcement not exceeding four lines.

**Notice to Advertisers and Subscribers.**

All Advertisements, Orders for Copies of THE HOSPITAL and Business Communications should be addressed to **The Manager, NOT TO THE EDITOR,** THE HOSPITAL, 428, Strand, London, W.C.
The Cost of Subscription, post free, is as follows:—Per week, 2½d.; per quarter, 2s. 8d.; per half-year, 5s. 3d.; per annum, 10s. 6d. Foreign:—Per Annum, 15s. 2d.; payable, in all cases, in advance.

### NOTICE TO CORRESPONDENTS.

All MS., Letters, Books for Review, and other matters intended for the Editor should be addressed THE EDITOR, THE LODGE, PORCHESTER SQUARE, LONDON, W.
The Editor cannot undertake to return rejected MS., even when accompanied by stamped directed envelope.

**"Burdett's Hospital Annual," 1895.**

READY NEXT WEEK.
Sixth year of publication. 950 pp. Scarlet cloth, gilt lettered. Price 5s. A most complete guide to British, Colonial, Indian, and American Hospitals, Dispensaries, Nursing and Convalescent Institutions, and Asylums. A few copies of the "Annual" for 1894 may still be obtained on application to the Publishers, THE SCIENTIFIC PRESS (Limited), 428, Strand, London, W.C.

**NOTICE.—Vol. XVII. of "The Hospital" (October, 1894, to April, 1895), in handsome cloth binding, is now on sale at the Office of this Journal. Price 6s. Cases for binding the Numbers contained in this Volume, 1s. 6d. Index to Vol. XVII., 2½d., post free.**
**The Monthly Part for June is now ready, price 6d.; by post, 8d.**

### SCRAPS AND GLEANINGS.

Russian medical literature is now circulated in thirty-eight journals.

The West Riding Sanitary Committee have found that the district of Rawdon is without means of isolation for cases of infectious disease. A committee has now been appointed to look for a site on which a hospital can be erected.

A decided increase in the amount of work done at the Glasgow Ear Hospital is reported for the last year. The directors of the institution will be in a position to extend their operations and they hope to receive the necessary funds for this purpose.

The report of the Lydney Cottage Hospital shows that 25 in-patients have received attention during the year, and that a balance of £35 is being carried forward on the finance accounts.

The opening of the new hospital at City Poorhouse, Aberdeen, took place last week, and, though not completely finished, the hospital will almost immediately be ready for occupation.

The report of the North Charitable Infirmary at Cork is a highly creditable one to the institution, and the committee are able to state that financial matters are in a fairly good condition. The new wing in connection with the hospital is soon to be opened.

Alterations and improvements of the Devon and Exeter Hospital are to be commenced immediately, and the committee are earnestly appealing to the generosity of the public for their practical support in raising money sufficient to cover the cost of the same.

The final ultimatum of the Liverpool City Council as to their financial aid to be given towards the New Northern Hospital scheme is fixed at £15,000, a sum equal to the utmost amount they have ever given to any other hospital of equal usefulness in Liverpool.

We learn that 2,509 bodies were cremated in Italy last year. Italy was one of the countries to lead this practice, but the Church having assumed a certain hostile attitude towards it, cremation does not show any marked increase as opposed to earth burial.

Her Majesty paid a visit to the camp of the Aberdeen Volunteer Medical Staff Corps last week, at Birkhall, near Balmoral. The Queen drove along the lines evincing great interest in the men who were lying bandaged and tied upon the stretchers as if wounded.

Under the Isolation Hospitals Act an inquiry was quite lately held by a committee of the County Council at Derby respecting a petition from the Alvaston Urban District Council for the establishment of an isolation hospital for the Alvaston and Shardlow Rural Districts.

The State Board of Health of Nebraska have constituted themselves judges of local dentistry, and dental practise will in future be governed by the same. This law, as a contemporary remarks, is little but a copy of the American Medical Practice Acts, extended to dentists.

A special laboratory is to be erected in Paris for bacteriological research, especially with the view of facilitating examination into and study of the bacillus of diphtheria. The Municipal Council of the French capital has guaranteed a sum of 10,000 francs towards this object.

A curious story comes to us from Kansas City. Five medical students have been arrested on the charge of grave-robbing, they being taken in the act. This robbery, which is of a somewhat new order, was perpetrated by members of the University Medical College of the city.

The project of bringing the University of Cambridge and Addenbrooke's Hospital into closer connection was carried a step nearer a definite issue at the last quarterly meeting of the governors, when it was agreed by a small majority to appoint a committee of governors to confer with the Vice-Chancellor and his assessors.

At a late meeting of the Thanet Joint Hospital Board it was finally agreed that the architects should be instructed to prepare plans and estimates for a new infectious hospital at a cost not exceeding £12,000, such plans to be subject to the approval of the Local Government Board. A loan is to be raised for carrying on the work.

Among the contributions towards the Derbyshire Royal Infirmary received in the past half-year, are £250 from Mr. Benjamin Stretton (late of Derby), £120 from His Grace the Duke of Portland, £63 from Lady and Miss Burdett. A large number of medical works have been presented to the infirmary library by Dr. Ogle, the hon. consulting physician.

In the first annual report of the Ashby Cottage Hospital we note that not only have the expenses of starting the hospital (which are necessarily heavy) and the cost of the patients been paid out of the annual subscriptions, but that there was at the end of the financial year a balance of £65. This is a matter for congratulation to the managers of the hospital.

The annual meeting of the Hospital for Women, Soho Square, has just been held. On an average about 43 beds are occupied during the year. The committee entertain hopes of extending the sphere of this institution, which is already one of most extreme usefulness. If money was forthcoming additional and adjoining premises would be purchased for this object.

The difficulties of centralising hospital accommodation for the isolation of infectious disease is no new phenomena; Swindon again brings these difficulties before our eyes. A County Council inquiry has quite recently been held on this subject, and an opposition presented from Gorse Hill residents against further increase of the area for the existing fever hospital placed there.

Mr. R. S. Hudson, the well-known soap manufacturer, sets a most praiseworthy example by supplying drinking troughs for dogs at his own expense outside grocers' shops all over the country. They are made of iron and white enamel, and already number 25,000. Lovers of animals could fitly show their appreciation of the kindly thought by seeing that none but Hudson's soaps were used for the canine toilet.

The total number of beds available at Carrington Convalescent Hospital, at Camden, Australia, is 95. During 1894 there were 977 patients. The average annual cost of each occupied bed was £34 8s. 6d. This report shows a considerable increase of work with a diminished cost per bed. The total expenditure was £2,569, and towards this 97 contributing patients paid £175 18s 8d. £1,082 14s. 9d. was the result of subscriptions and donations. Financially, owing to the receipt of several legacies, this hospital has a credit balance.

### THE STUDENT OF MEDICINE.

### APPOINTMENTS.

G. J. Arnold, L.R.C.P., M.R.C.S., appointed House Surgeon to St. Thomas's Hospital (extension). G. B. C. Blount, L.R.C.P., M.R.C.S., appointed Clinical Assistant in the Special Department for Diseases of the Ear at St. Thomas's Hospital. F. J. Brakenridge, L.R.C.P., M.R.C.S., appointed Non-Resident House Physician to St. Thomas's Hospital (extension). G. J. Branson, L.R.C.S., L.R.C.P.Edin., L.S.A., B.A.Lond., appointed House Surgeon to the Queen's Hospital, Birmingham. H. T. Brickwell, M.R.C.S., L.S.A., appointed Medical Officer and Public Vaccinator for the Chigwell District of the Epping Union. G. Candler, B.A.Cantab., L.R.C.P., M.R.C.S., appointed Junior Obstetric House Physician to St. Thomas's Hospital. F. W. Clark, M.B.Durh., D.P.H.Camb., M.R.C.S., L.R.C.P.Lond., Medical Officer of Health for the Borough and Port of Lowestoft, appointed (by the Crown) Health Officer for Hong Kong. G. J. Coxford, B.A.Oxon., L.R.C.P., M.R.C.S., appointed Clinical Assistant in the Electrical Department of St. Thomas's Hospital. E. G. C. Daniel, L.R.C.P., M.R.C.S., B.A.Cantab., appointed Resident House Physician to St. Thomas's Hospital. H. J. Davis, M.A.Cantab., L.R.C.P., M.R.C.S., appointed Assistant House Surgeon to St. Thomas's Hospital. W. E. Dixon, L.R.C.P., M.R.C.S., B.Sc.Lond., appointed Clinical Assistant in the Electrical Department at St. Thomas's Hospital. C. E. Dumbleton, M.A., M.D.Cantab., M.R.C.S.Eng., D.P.H., appointed Surgeon to the Dalby Hospital, Queensland. W. D'Este Emery, M.R.C.S.Eng., L.R.C.P.Lond., B.Sc.Lond., appointed House Physician to the Queen's Hospital, Birmingham. Dr. Fleming, appointed Medical Officer for the Newbridge Dispensary District. Dr. Fletcher, appointed Medical Officer for the Castle Donington District of the Shardlow Union. G. G. Genge, L.R.C.P., M.R.C.S., appointed Clinical Assistant in the Special Department for Diseases of the Ear at St. Thomas's Hospital. T. Greer, M.D., appointed Medical Officer for the Second District of the Cambridge Union. A. W. Haines, L.S.A.Lond., B.Sc.Lond., appointed Obstetric and Ophthalmic House Surgeon to the Queen's Hospital, Birmingham. H. W. Harding, L.R.C.P., M.R.C.S., appointed House Surgeon to St. Thomas's Hospital (extension). J. W. Hayward, M.R.C.S.Eng., L.S.A., appointed Port Medical Officer for Whitstable. Dr. Jacobson, appointed Medical Officer of Health to the Kingston Urban District Council. T. J. P. Jenkins, M.R.C.S.Eng., L.R.C.P.Lond., appointed Medical Officer for the First South-Eastern District of the Freebridge Lynn Union. L. L. Jenner, M.A., M.B., B.Ch.Oxon, M.R.C.P., appointed Resident House Physician to St. Thomas's Hospital. J. W. Laver, L.R.C.P., M.R.C.S., appointed Non-resident House Physician to St. Thomas's Hospital (extension). S. R. Lister, M.R.C.S., L.R.C.P.Lond., appointed Medical Officer for the Fourth and Fifth Districts of the Wisbech Union. F. V. Milward, M.B., B.C.Cantab., L.R.C.P., M.R.C.S., appointed Clinical Assistant in the Special Department for Diseases of the Skin at St. Thomas's Hospital. W. E. Porter, M.D.Edin., M.R.C.S. Eng., appointed Medical Officer to the Printers' Almshouses. J. L. Prain, L.R.C.P., M.R.C.S., appointed Clinical Assistant in the Special Department for Diseases of the Throat at St. Thomas's Hospital (extension). W. Randall, L.R.C.P.Edin., M.R.C.S.

THE STUDENT OF MEDICINE—*continued.*

Eng., appointed Medical Officer of Health to the Maesteg Urban District Council. S. W. F. Richardson, M.B., B.S., B.Sc.Lond., L.R.C.P., M.R.C.S., appointed Senior Obstetric House Physician to St. Thomas's Hospital. Anthony Roche, M.R.C.P.I., appointed Professor of Medical Jurisprudence and Hygiene in the Catholic University Medical School, Dublin. A. E. Russell, M.B.Lond., L.R.C.P., M.R.C.S., appointed House Surgeon to St. Thomas's Hospital (extension). Allan L. Saunders, M.R.C.S. Eng., L.R.C.P.Lond., appointed Medical Officer and Public Vaccinator for the Eastern District, and Surgeon to the Workhouse of the Freebridge Lynn Union. P. J. A. Seccombe, M.A. Cantab., L.R.C.P., M.R.C.S., appointed Clinical Assistant in the Special Department for Diseases of the Throat at St. Thomas's Hospital. H. R. Sedgwick, B.A.Cantab., L.R.C.P., M.R.C.S., appointed Clinical Assistant in the Special Department for Diseases of the Skin at St. Thomas's Hospital. William C. Steen, M.D., &c., R.U.I., appointed Honorary Assistant Physician to the Belfast Hospital for Sick Children. W. G. Stone, M.A., M.B., B.Ch.Oxon., L.R.C.P., M.R.C.S., appointed Assistant House Surgeon to St. Thomas's Hospital. Charters J. Symonds, M.S.Lond., F.R.C.S.Eng., appointed Consulting Surgeon to the London and South-Western Railway Servants' Orphanage. R. Fox Symons, L.R.C.P., M.R.C.S., appointed House Surgeon to St. Thomas's Hospital (extension). J. F. Tabb, M.R.C.S.Eng., L.S.A., appointed Medical Officer for the North-Eastern District of the Greenwich Union. H. G. Toombs, L.R.C.P., M.R.C.S., appointed Senior Ophthalmic House Surgeon to St. Thomas's Hospital. F. C. Wallis, M.B., B.C.Cantab., F.R.C.S., appointed Surgeon to the Out-patients at Paddington Green Children's Hospital. A. M. Watkins, L.R.C.P Lond., M.R.C.S.Eng., appointed Medical Officer for the Workhouse and the Whitchurch District of the Whitchurch (Salop) Union. B. Watts, M.R.C.S.Eng., L.R.C.P. Lond., appointed House Surgeon to the Children's Hospital, The Wicker, Sheffield. E. F. Weston, M.R.C.S.Eng., appointed Medical Officer for the Seighford District of the Stafford Union. J. F. Wright, L.R.C.P.Lond., M.R.C.S.Eng., appointed Medical Officer for the West Bolton District of the Bolton Union.

### VACANCIES.

The following vacancies are announced this week, the amount of salary in each case being given after the name of the institution:

#### Resident Medical Officers.

City of London Hospital for Diseases of the Chest, Victoria Park, E.—House Physician. Board, residence, and allowance for washing provided. Appointment for six months. Applications to the Secretary, at the office, 24, Finsbury Circus, E.C., by June 13th.

Hospital for Sick Children, Great Ormond Street, Bloomsbury, W.C.—House Surgeon to the Out-patients. Appointment for six months, but eligible for re-election for a further six months. Salary 25 guineas. Applications to the Secretary by June 18th.

Durham County Asylum.—Pathologist and Junior Resident Medical Officer. Salary £100 per annum. Applications to the Superintendent, Durham County Asylum, Winterton, Ferryhill, by June 8th.

Kensington Dispensary.—Resident Medical Officer; unmarried; under 35 years of age, and doubly qualified. Salary £125 per annum, with furnished apartments, coals, gas, and attendance. Applications to the Honorary Secretary, Mr. Frederick Leach, 7, Stanford Road, Kensington Square, by June 12th.

London County Council.—Pathologist to the London County Asylums. Salary £700 per annum, and travelling expenses. Applications (on forms provided), endorsed "Applications for Pathologist," to R. W. Partridge, Clerk of the Asylums Committee, 21, Whitehall Place, S.W., by June 17th.

Manchester Royal Infirmary.—Resident Medical Officer; not under 25 years of age, unmarried, and doubly qualified. Appointment for one year, but eligible for re-election. Salary £150 per annum, with board and residence. Applications to the Chairman of the Board by June 22nd.

North-Eastern Hospital for Children, Hackney Road, Shoreditch, N.E.—House Physician. Appointment for six months, and after the expiration of this term the House Physician will be required, if eligible, to serve as House Surgeon for a further period of six months. Must possess a medical and surgical qualification. Salary as House Physician at the rate of £60 per annum, and as House Surgeon at the rate of £80 per annum. Applications and testimonials to T. Glenton-Kerr, Secretary, City Office, 27, Clement's Lane, Lombard Street, E.C., by June 11th.

Parish of Birmingham Workhouse Infirmary.—Resident Assistant Medical Officer; doubly qualified. Salary £100 per annum, with furnished apartments, rations (no alcoholic liquors), coals, washing, and attendance. Applications, on forms provided, to Walter Bowen, Clerk to the Guardians, Parish Offices, Birmingham, by June 8th.

Radcliffe Infirmary, Oxford.—House Physician. Appointment for six months. Salary at the rate of £60 per annum, with board and lodging. Applications, on printed forms provided, to be sent to the Secretary before June 19th.

Royal Ear Hospital, Frith Street, Soho, W.—House Surgeon. Appointment for six months. Honorarium £12 12s. Application to D. Murray, Honorary Secretary, by June 8th.

West London Hospital, Hammersmith Road, W.—House Physician and House Surgeon. Appointments for six months. Board and lodgings provided. Applications to R. J. Gilbert, Secretary-Superintendent, by June 19th.

#### Non-Resident Medical Officers.

Dental Hospital of London, Leicester Square.—Two Dental Surgeons. Candidates must be Licentiates of Dental Surgery of one of the licensing bodies recognised by the General Medical Council. Applications and testimonials to J. Francis Pink, Secretary, by June 10th.

Trinity College, Dublin.—Lecturer in Pathology. Applications to the Registrar of the University by June 15th.

Vestry of Lambeth.—Medical Officer of Health; not less than 25 or more than 45 years of age. Salary £700 per annum, such salary to include the cost of a carriage to be provided by the officer. Applications, endorsed "Application for Appointment of Medical Officer," to Henry J. Smith, Clerk to the Vestry, Vestry Hall, Lambeth, by June 11th.

